# A Two-Year Retrospective Comparison of Clinical Outcomes Between Open Release and Percutaneous Release of the Trigger Finger

**DOI:** 10.7759/cureus.99798

**Published:** 2025-12-21

**Authors:** Saran Malisorn

**Affiliations:** 1 Orthopaedics, Naresuan University, Phitsanulok, THA

**Keywords:** a1 pulley release, dash score, open surgery, percutaneous trigger finger release, trigger finger

## Abstract

Background: Trigger finger is often found in adults, accompanied by pain, swelling, and limited movement. If conservative treatment fails, surgical release of the A1 pulley will be performed. Percutaneous trigger finger release is a new surgery method with rapid recovery that includes a good outcome, but there is also more reluctance to use this standard technique than open surgery. This article, therefore, presents a comparison of the long-term outcomes of both methods across different factors, similarities, and patient groups. The study hypothesized that the percutaneous trigger finger release had a far better result than open surgery.

Study methodology: Between 2012 and 2022, a total of 166 patients with trigger finger treated with either open A1 pulley release (n = 83) or percutaneous release (n = 83) were enrolled in the study. The outcomes were evaluated in both short-term and long-term periods. The disabilities of the arm, shoulder, and hand questionnaire scores (DASH) and visual analog scale (VAS) scores before and after surgery were compared between the two groups for bleeding, digital nerves, artery injury, inability to bend the finger, and others at diﬀerent points at a two-year follow-up.

Results: Both groups of patients were statistically similar in age and gender. There was no significant difference in the number of DASH scores and VAS scores for pain between the two groups before surgery. However, the group that underwent percutaneous trigger finger release had a very low differentiation at three months. Pain scores and other variables returned to normal with no difference between the groups at two-year long-term follow-up. No significant differences were found in complications, such as traumatic pain, digital nerves, and arterial injuries, between the groups.

Conclusion: The study found that percutaneous trigger finger release in trigger finger patients was more practical than traditional open surgery in supporting their long-term clinical outcomes. This technique results in less pain and less bleeding, and the complications were not that different from traditional open surgery in two years.

## Introduction

Trigger finger is most commonly found in hand disorders. This abnormality causes pain, a clicking sound, or obstruction or sticking while moving the fingers, which makes it difficult to carry out daily activities. Although the exact cause is unknown, the main cause of trigger finger stimulation is inﬂammation and contraction of the A1 pulley. This results in the jammed movement of flexor tendon lines and thickening of fibrous formations on the surface of the tendon sheath. Histological studies have revealed an additional layer of structure that includes the chondroid metaplasia [1]. This condition usually occurs in people over 40 years of age.

According to previous research, women have a six times higher likelihood of developing trigger finger compared to men [2]. If left untreated, this condition could result in the fingers becoming stuck, difficulty in stretching them in and out, and persistent pain. The condition is commonly associated with the thumb and index finger of the dominant hand [2]. Conservative treatment of trigger finger includes splinting, corticosteroid injections, and other supportive methods. Trigger finger can be treated in diﬀerent ways, depending on the stage of the disease. For patients in the early stages of trigger finger, utilizing a finger splint throughout the day and night, engaging in self-physiotherapy, and employing various painkillers such as anti-inflammatory drugs, along with triamcinolone injections into the affected area, are suitable treatment options and a favorable choice for patients.

A1 pulley release is indicated in more advanced stages of trigger finger and can be performed using either open surgery or a percutaneous technique [3]. Open surgery has been performed for many years and is 97% effective. However, it can cause surgical complications, such as pain after the procedure, risk of infection, increased recovery time from movement, nerve injury, and scarring [4,5]. Percutaneous trigger finger release is another popular alternative method with a reported success rate of 74-94% [6]. The percutaneous trigger finger release method has been found to reduce injuries and speed up recovery time. Nevertheless, there is a possibility of nerve and artery damage as well as incomplete surgery [7]. Clinical studies that compare outcomes in patients after surgical treatment are of great importance in this regard. They can help professionals choose and practice the method most suitable for them. Although the results of open surgery and percutaneous trigger finger release after three months (short-term) and two-year (long-term) follow-up [8,9] were previously published, limited studies reported results by comparing similar groups of patients. This retrospective study involved comparing the two-year clinical outcomes of standard open surgery and percutaneous trigger finger release with the assumption that percutaneous trigger finger release techniques provide better results than open surgery does.

## Materials and methods

Study design and population

This is a retrospective group study conducted on patients who underwent open surgery or percutaneous trigger finger release at Naresuan University Hospital between 2012 and 2022. The inclusion criteria were adults aged ≥18 and were rated 2-5 according to the improved Quinnell grading system [3]. In contrast, patients with temporary trigger fingers who underwent steroid injections were given the treatment ≤ eight weeks before the study. Patients with previous trigger finger surgery, tendon injury or fracture of the finger or palm in the affected area, degenerative arthritis, gout affecting the finger joints, rheumatoid arthritis or other inflammatory rheumatic diseases, and connective tissue diseases were excluded from this study. In addition, patients with a history of drug allergy to nonsteroidal anti-inﬂammatory drugs, peptic ulcer disease or gastrointestinal bleeding, asthma, a history of chronic liver or chronic biliary tract disease, and kidney disease were also excluded from this study. This study is in accordance with the Declaration of Helsinki and was approved by the Ethics Committee of Naresuan University (P3-0013/2023, COA No.062/2023). Participants gave verbal consent based on the nature of the study, and this is authorized by the Institutional Review Committee.

Sample size

In the determination of the group size used in the study, the two independent proportions were calculated by using the two-sided test method as previously described in the referenced article [10]. The proportion of open surgery (p1 = 0.97) was considered in previous studies [11], while percutaneous trigger finger release (p2) was defined at 0.84 with an alpha-type error rate of 5% and statistical power of 80%. The required sample size was 83 patients in each group.

Surgical techniques

Both surgical methods were performed according to the standard aseptic technique in the hospital's orthopedic department. First, the location was indicated and marked. Afterward, the injection site was administered with a 1 cc injection of 1% xylocaine. In open surgery, a transverse incision of 1-1.5 cm was performed. Once the A1 pulley was separated, the flexor digitorum profundus tendon and flexor digitorum communis tendon could be fully stretched, allowing for improved mobility. The wound was sutured with nylon 4/0 and closed to prevent infection. Surgery on another group of patients treated with percutaneous trigger finger release was performed using a technique based on the principles described by Phithakrat [12], but adapted to use a No. 15 blade instead of an 18-gauge needle, as detailed below. Stretching the patient's fingers to their utmost extent resulted in the blood vessels and nerves of the finger shifting to the side and the flexor tendon rising closer to the skin. The tip of blade No. 15 was then inserted perpendicularly through the skin at the position of A1. The blade tip was shifted to a speciﬁed extent of about 5-8 mm to cut the A1 pulley. When the blade tip cut through the transverse fiber, it caused a sense of separation of the A1 pulley, and this feeling indicated that the procedure was complete.

Then, the operating surgeon performed the test to confirm that the surgery was successful and that the finger was no longer completely locked by stretching and clenching the finger quickly. Testing with this process was repeated about 10 times, and the wound was covered with gauze. Following surgery, the patients were discharged and returned home without requiring hospital admission. In addition, the patients were provided with instructions on wound care, proper behavior, and painkillers and antibiotics. Subsequently, follow-up appointments were scheduled at one week, six weeks, three months, 12 months, and 24 months, respectively, to assess wound healing, postoperative discomfort, potential complications, and recurrence rate.

Data collection

This research study involved the authorized collection of data from the medical records of patients in the hospital. Various outcomes, including DASH and VAS scores [13-16], postoperative outcomes, such as bleeding, damage to digital nerves and arteries, facial pain scores, and other outcomes, were recorded in the logbook as mentioned earlier [8].

Statistical analysis

Patient demographic data included frequency, percentage, mean values, and standard deviations. Continuous variable comparisons between two groups were performed using Student's t-test, while analysis of covariance was conducted using the chi-square test, considering a statistically significant P-value < 0.05. Statistical analysis was performed using IBM SPSS Statistics version 17 software (IBM Inc., Chicago, IL, USA).

## Results

From the basic characteristics of the patients in the study, out of a total of 166 patients, the majority were women (72.23%). The number of patients with trigger fingers in the open surgery group and the percutaneous trigger finger release group, when compared, showed no statistically significant difference. The results regarding the gender of patients who had trigger fingers and underwent surgery in both groups were also insignificant. The age of patients in both groups also showed no statistically significant difference. Based on the data on the age of those patients, it is evident that there were more patients below the age of 60 years old than above 60 years old. There was a significant difference in the side of the affected hand between the two groups. The severity of trigger finger stages in both surgery groups of patients had no statistically significant difference. However, this study found that the middle and ring fingers were the most symptomatic fingers.

All patients had a single finger diagnosed with trigger finger, and the duration of trigger finger symptoms was not statistically significant between the two groups. About 25% of patients are farmers and government officials, as shown in Table 1.

**Table 1:** Baseline characteristics of the patient. *Diabetes mellitus with controlled fasting blood sugar (FBS) (<126 mg/dl). COPD: chronic obstructive pulmonary disease.

Characteristics	Open (n = 83)	Percutaneous (n = 83)	p-value
Gender, n (%)			0.72
Male	24 (28.92)	22 (26.51)	
Female	59 (71.08)	61 (73.49)	
Age, n (%)			0.48
<60	58 (69.88)	62 (74.70)	
>60	25 (30.12)	21 (25.30)	
Hand side, n (%)			<0.01
Left	11 (13.25)	42 (50.60)	
Right	72 (86.75)	41 (49.40)	
Career			0.90
Agriculture	15 (18.07)	12 (14.46)	
Government	12 (14.46)	14 (16.87)	
Personal officer	9 (10.84)	8 (9.64)	
Trade business	7 (8.43)	7 (8.43)	
Unemployed	3 (3.61)	6 (7.23)	
Other	37 (44.58)	36 (43.37)	
Grade, n (%)			0.36
3	39 (46.99)	31 (37.35)	
4	26 (31.33)	26 (31.33)	
5	10 (12.05)	18 (21.69)	
6	8 (9.64)	8 (9.64)	
Affected digit (%)			0.71
Index finger	18 (21.69)	24 (28.92)	
Middle finger	30 (36.14)	29 (34.94)	
Ring finger	28 (33.73)	23 (27.71)	
Little finger	7 (8.43)	7 (8.43)	
Finger affected, n (%)			
1	83 (100)	83 (100)	NA
Duration of symptom (months)	3.14 (1.54)	2.80 (1.45)	0.14
Activity, n (%)			
Hand overuse	83 (100)	83 (100)	NA
Underlying diseases, n (%)			
Diabetes mellitus*	21 (25.30)	24 (28.92)	0.60
Hypertension dyslipidemia	15 (18.07)	16 (19.28)	0.84
Dyslipidemia	4 (4.82)	0 (0)	0.04
Asthma	0 (0)	1 (1.20)	0.31
COPD	2 (2.41)	3 (3.61)	0.65
Surgery outcome, n (%)			
Trigger finger release	83 (100)	83 (100)	NA

Preoperative visual analog scale (VAS) and disabilities of the arm, shoulder, and hand (DASH) pain score information

The preoperative VAS scores for trigger finger pain did not differ significantly between the open surgery and percutaneous release groups (6.79 ± 1.26 vs 7.03 ± 1.54; p = 0.27, which is not statistically significant), as shown in Table 2.

**Table 2:** Summary of findings before the trigger finger release. VAS: visual analog scale; DASH: disabilities of the arm, shoulder, and hand.

Variables	Before		p-value
	Open	Percutaneous	
VAS score (mean ± SD)	6.79 ± 1.26	7.03 ± 1.54	0.27
Faces Pain Scale score (mean ± SD)	3.04 ± 0.81	3.40 ± 0.88	<0.01
Grade, n (%)			0.70
1	40 (48.19)	41 (49.40)	
2	34 (40.96)	30 (36.14)	
3	9 (10.84)	12 (14.46)	
DASH score (mean ± SD)	31.02 ± 8.45	31.80 ± 10.24	0.59

From this study, it was found that the differences in preoperative pain between the two groups were statistically significant. Each group, when scored based on facial expression pain scores, showed similar levels of severity in the trigger finger range and DASH scores before undergoing surgery.

Clinical outcomes for postoperative follow-up in both groups

Both groups of patients underwent successful and complete A1 pulley release surgeries. However, the open surgical group reported a case of nerve injury in one patient who experienced symptoms of trauma to the digital nerve. In the first week of follow-up, the rate of postoperative bleeding was higher in the open surgery group than in the percutaneous trigger finger release group (30.12% vs 3.61%). Similarly, the DASH score for the period of three months after surgery in the open trigger finger surgery was higher compared to those who underwent percutaneous trigger finger release. This is consistent with the VAS score of both groups. The open trigger finger surgery group also had a higher score value compared to the percutaneous trigger finger release group, as shown in Table 3.

**Table 3:** Summary of the clinical outcomes in short-term follow-up (three months). VAS: visual analog scale; DASH: disabilities of the arm, shoulder, and hand. *Significant <0.05.

Variables	One week		Six weeks		Three months	
	Open	Percutaneous	Open	Percutaneous	Open	Percutaneous
Grade 0	83 (100)	83 (100)	83 (100)	83 (100)	83 (100)	83 (100)
Bleeding	25 (30.12)*	3 (3.61)*	0 (0)	0 (0)	0 (0)	0 (0)
Digital nerve injury	1 (1.20)	0 (0)	0 (0)	0 (0)	0 (0)	0 (0)
Digital artery injury	0 (0)	0 (0)	0 (0)	0 (0)	0 (0)	0 (0)
DASH score	8.3 ± 8.26	8.63 ± 10.01	0 (0)	0 (0)	0 (0)	0 (0)
Wound pain	83 (100)	80 (96.39)	0 (0)	0 (0)	0 (0)	0 (0)
Inability to flex the finger	8 (9.64)	4 (4.82)	0 (0)	0 (0)	0 (0)	0 (0)
VAS score			1.02 ± 0.68*	0.43 ± 0.56*	0.10 ± 0.31*	0 ± 0*
Faces Pain Scale score			0.48 ± 0.50*	0.13 ± 0.34*		

The overall pain scores, including other variables, returned to normal without any statistically significant differences between the groups in the long term (>3 months; Table 4).

**Table 4:** Summary of the clinical outcomes in long-term follow-up (>3 months). VAS: visual analog scale; DASH: disabilities of the arm, shoulder, and hand.

Variables	One year		Two years	
	Open	Percutaneous	Open	Percutaneous
Grade 0	83 (100)	83 (100)	83 (100)	83 (100)
Bleeding	0 (0)	0 (0)	0 (0)	0 (0)
Digital nerve injury	0 (0)	0 (0)	0 (0)	0 (0)
Digital artery injury	0 (0)	0 (0)	0 (0)	0 (0)
DASH score	0 (0)	0 (0)	0 (0)	0 (0)
Wound pain	0 (0)	0 (0)	0 (0)	0 (0)
Inability to flex the finger	0 (0)	0 (0)	0 (0)	0 (0)
VAS score	0 (0)	0 (0)	0 (0)	0 (0)
Faces Pain Scale score	0 (0)	0 (0)	0 (0)	0 (0)

**Figure 1:**
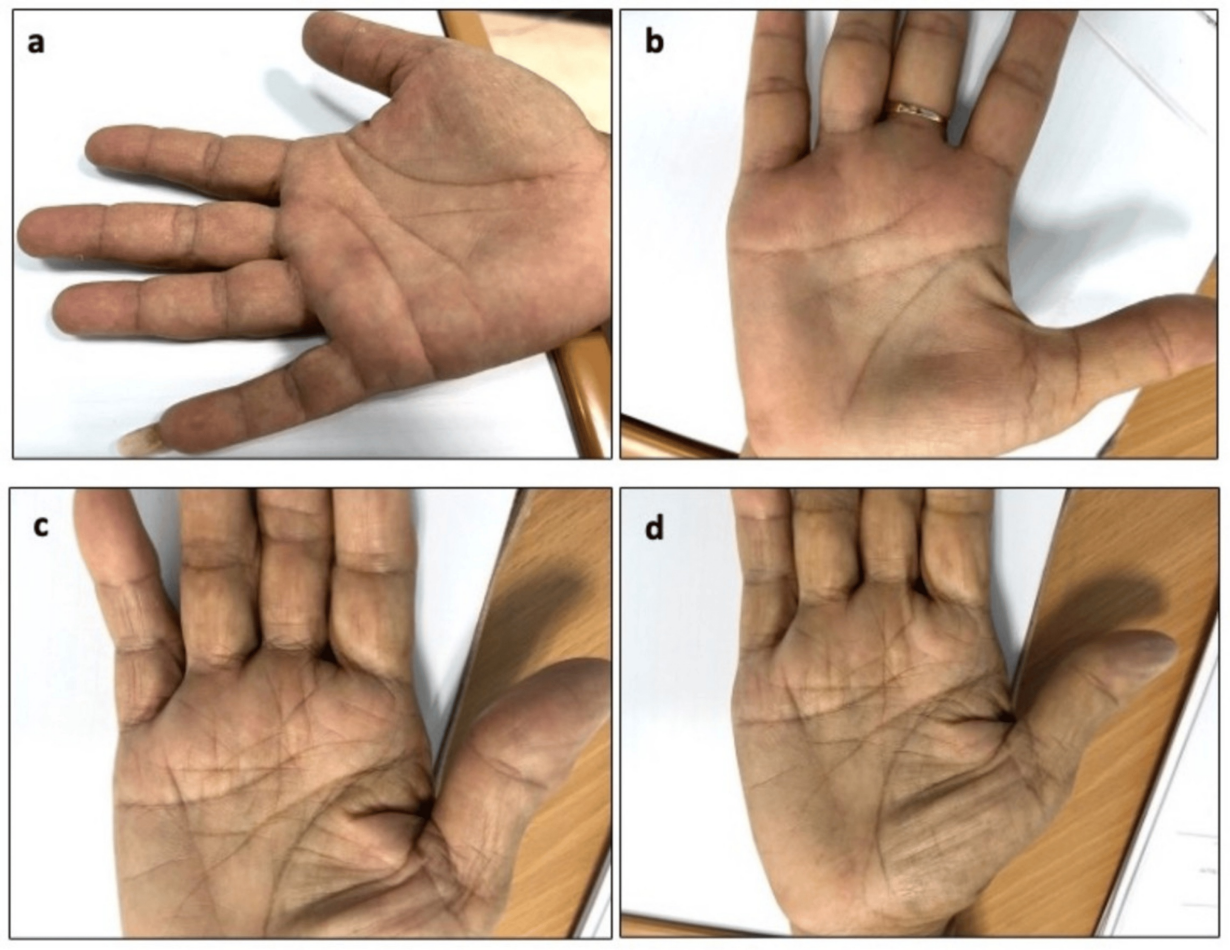
Representative pictures of the hand at short-term (three months) and long-term (two years) follow-up after either surgery. (a) Three months and (b) two years after the percutaneous surgery; (c) three months; and (d) two years after the open surgery.

There was no statistically significant difference between short-term (three months) and long-term (two years) follow-up after both forms of surgery (Figure 1).

## Discussion

The aim of this retrospective study was to compare short-term and long-term outcomes of traditional open trigger finger surgery with those of percutaneous trigger finger release. The study spanned a period of two years. The two groups of patients were categorized by gender and age. During the three-month follow-up, the group that underwent percutaneous trigger finger release exhibited a normal DASH score. However, some patients still experienced challenges in flexing their fingers. In addition, all trigger finger treatments were recorded without any complications such as peripheral nerve injury. Percutaneous trigger finger release has been shown to produce superior outcomes compared to open surgery and is also a safer procedure, as previously mentioned.

According to a previous retrospective study, trigger finger disease was found to be more prevalent in women compared to men [17,18]. In this study, 72.28% of patients were younger than 60 years, and this disease was more common in the age group 40-60 years [17]. The association of gender and age in trigger finger disease is unclear. In general, repeated use fingers are likely to cause the malfunction. According to various studies on the topic, 60-70% of patients with trigger fingers experience the condition in their middle and ring fingers.

According to studies on trigger finger, 68.07% of patients have been affected in the right hand. A similar finding was reported earlier [18,19]. In comparable studies, approximately 70% of patients with trigger finger in stages 3 and 4 were observed. This means that the patients had intermittent movement and trigger finger symptoms, but they were able to recover on their own.

In our research, the VAS score did not differ significantly between patients who underwent percutaneous release and those who underwent open surgery. This differs from some previous reports, which have reported better pain outcomes following percutaneous release [20]. One possible difference is how patients were chosen and the types of comorbidities they had. Similarly, both groups used similar analgesic preoperative life-support systems, so this is not likely to be a major consideration. In addition, retrospective cohort studies usually have very few subjects and may have overly limited statistical technology to detect small differences in pain scores.

According to the results of the study, the differences in facial pain scores and VAS scores between the two groups can be attributed to the patients' perception and occupation, as well as their advancing age, literacy, and cognitive ability. There is a possible explanation for the significant difference in the base of the facial pain score between the groups. It is claimed that both the level of facial pain scores and VAS scores are suitable for evaluating acute pain after surgery [21].

After one week of surgery, the DASH score's base did not differ between traditional open surgery and percutaneous trigger finger release. It can be observed that there is no statistically significant difference in the postoperative outcomes at three months when compared to the results within the first week.

In our cohort, DASH scores improved in both groups at one week and at three months after surgery, indicating a marked functional recovery over time. Previous studies have reported high success rates and low complication rates with percutaneous trigger finger release [22]. In our study, we did not observe major complications in the percutaneous group; however, this finding should be interpreted with caution due to the limited sample size and the retrospective design, and it cannot be taken as proof of 100% effectiveness or superiority in all aspects.

This study has several limitations. First, retrospective studies may result in bias. Second, exclusion of patients with congenital diseases may affect current findings. Third, the measurement of parameter results is limited. Fourth, patients who have thumb trigger fingers could not be included in this study due to the higher risk of peripheral neurovascular injury [23]. Therefore, the comparison between the patient’s thumb and finger is important to enhance the impact of such research results.

## Conclusions

This study has demonstrated that percutaneous trigger finger release for trigger finger treatment has no statistically significant difference to traditional open surgery treatment based on patients' short- and long-term clinical outcomes. The results of this study can help the use of percutaneous trigger finger release to achieve better results in a short period and at low risk. Nevertheless, it has been suggested that, except for patients with severe trigger finger, percutaneous trigger finger release should be considered in conjunction with other guidelines.
